# Experience of relatives in the first three months after a non-COVID-19 Intensive Care Unit discharge: a qualitative study

**DOI:** 10.1186/s12875-022-01720-z

**Published:** 2022-05-05

**Authors:** Matteo Danielis, Stefano Terzoni, Tamara Buttolo, Chiara Costantini, Tommaso Piani, Davide Zanardo, Alvisa Palese, Anne Lucia Leona Destrebecq

**Affiliations:** 1 Doctoral Programme in Public Health Sciences, Department of Clinical Sciences and Community Health, University of Milan, Via Vanzetti 5, 20133 Milan, Italy; 2 School of Nursing, Department of Medical Sciences, University of Udine, Viale Ungheria 20, 33100 Udine, Italy; 3 School of Nursing, San Paolo Teaching Hospital, Via Ovada, 26, 20142 Milan, Italy; 4 Department of Anesthesia and Intensive Care, Udine Teaching Hospital, Piazzale Santa Maria della Misericordia, 15, 33100 Udine, Italy; 5 School of Nursing, Department of Biomedical Sciences for Health, University of Milan, Via Pascal, 36, 20133 Milan, Italy

**Keywords:** Coronavirus disease 2019, Follow-up, Intensive care unit, Qualitative research, Family

## Abstract

**Background:**

The novel coronavirus brought Intensive Care Units (ICUs) back to their past when they were closed to family members. The difficulties of family caregivers encountered after the ICU discharge might have been increased during the coronavirus disease 2019 (COVID-19) pandemic. However, no traces of their experience have been documented to date. The objective of this study is to explore the everyday life experience of relatives in the first three months after a non-COVID-19 ICU discharge.

**Methods:**

A descriptive qualitative study was conducted in 2020–2021. Two Italian general non-COVID-19 ICUs were approached. Follow-up telephone interviews were conducted three months after the ICU discharge. The study has been conducted according to the COnsolidated criteria for REporting Qualitative research principles.

**Results:**

A total of 14 family members were interviewed. Participants were mostly females (*n* = 11; 78.6%), with an average age of 53.9 years. After three months of care of their beloved at home, relatives’ experience is summarised in three themes: “Being shaken following the ICU discharge”, as experiencing negative and positive feelings; “Returning to our life that is no longer the same”, as realising that nothing can be as before; and “Feeling powerless due to the COVID-19 pandemic”, given the missed care from community services and the restrictions imposed.

**Conclusions:**

Relatives seem to have experienced a bilateral restriction of opportunities – at the hospital without any engagement in care activities and their limited possibility to visit the ICU, and at home in terms of formal and informal care.

**Supplementary Information:**

The online version contains supplementary material available at 10.1186/s12875-022-01720-z.

## Background

A large number of Intensive Care Unit (ICU) patients are ultimately discharged to home with different degrees of functional dependence and cognitive damages requiring continuous care. In a large study performed by Gayat et al. (2018) including a cohort of 2,087 patients in France and Belgium, ICU mortality was 22%, and 333 patients (21%) died in the year after the ICU discharge [1]. Similarly, Detsky et al. (2017) conducted a prospective cohort study in five medical and surgical ICUs in Pennsylvania (US) measuring post-intensive care syndrome at six months after discharge. Of the 303 enrolled patients, 72 (23.8%) died in hospital and 169 (55.8%) were alive at the six-month follow-up. Among survivors, 121 (71.3%) were able to mobilise and 138 (81.9%) were able to toilet, while normal functional cognition was reported for 105 (62.4%) [2].

Relatives have a significant role in patient recovery as they are called to provide physical and emotional support. The impact of family care has been investigated in both quantitative [3] and qualitative research [4–6]. Primarily, after ICU discharge, relatives have been reported to be tired, given that most of the patient’s activities in daily living have to be assisted by hands-on care; moreover, relatives have been reported to perceive themselves as unready because the home environment needs to be rearranged and new competences and skills need to be acquired as well [5]. At the same time, family carers are optimistic as the patient’s little improvements trigger hope [5]. A review performed by Johnson et al. (2019) summarised the types of burden reported by relatives within a follow-up ranging from one to 53 months after ICU discharge as follows: anxiety (2–80%), depression (4–94%), and post-traumatic stress disorder (PTSD) (3–62%) [7]. In addition, lifestyle interferences (e.g. loss of employment and financial issues), and low health-related quality of life have also been frequently reported among relatives [8]. According to the increased body of evidence regarding the long-term implications on family relatives, the Critical Care Medicine Society has recently introduced the concept of Post-Intensive Care Syndrome-Family (PICS-F) reflecting their physical, cognitive, and mental burden [9]. In this context, a recent systematic review of 11 randomized clinical studies published between 2000–2019 addressing interventions for relatives of patients during their ICU stay (*n* = 6), after their ICU discharge (*n* = 4), or in both periods (*n* = 1), reported that proactive communication and provision of information seems essential for PICS-F management, although the lack of studies suggest the need to further expand this field of research [10].

In the last 15 years, ICU policies in Italy have been changed by improving visitors’ accessibility and by engaging family relatives in the care of patients during hospitalisation to increase their awareness of and readiness for the care required after the ICU discharge [11, 12]. The coronavirus disease 2019 (COVID-19) pandemic has brought the ICU back to its past when relatives were used to seeing their loved ones through the glass. With the intent of ensuring both patient and visitor safety, family hospital visiting has been suspended [13, 14]. The spread of the pandemic has also introduced substantial changes in the ICUs environments, increasing up to 50% or more in beds [15] and 33% of the nurses’ workloads [16], hiring also new staff to care for critically ill patients. As a consequence, some patients’ needs were compromised or missed, and their interactions with family members were significantly reduced [17].

However, hospitals are not the only care settings that have been affected by the pandemic, provision of community care has also been impacted. Lockdown measures have been reported as affecting patients – especially those with physical disabilities – and their relatives, by reducing family cohesion and preventing the delivery of the required healthcare support [18]. Relatives’ difficulties and challenges encountered after the ICU discharge have been largely documented [4–6, 8]; suggesting the need for smooth care transitions (e.g., in neurocritical care patient population [19]). However, to date, no research has documented the influence of the coronavirus pandemic on the relatives’ experiences after ICU discharge. Thus, the general purpose of this study was to explore the everyday life experience of relatives in the first three months after a non-COVID-19 ICU discharge.

## Methods

### Aim

The research question addressed was: ‘What are the everyday life experiences of relatives caring for a loved one at home, after the ICU discharge for non-COVID-19 issues, during the pandemic?’. According to the research question, the study’s aim was to explore and describe the experiences of a relative who has been facing day-to-day life during the first three months after a non-COVID-19 ICU discharge.

### Design

A descriptive qualitative study was conducted from August 2020 to January 2021 to discover how relatives make sense of their experiences [20, 21]. Methods and findings are reported here according to the COnsolidated criteria for REporting Qualitative research principles [22] (see Supplementary Table 1).

### Setting and participants

Patients and relatives were recruited in two general ICUs of an Academic Hospital in the North-East of Italy (> 1,000 beds). These two ICUs, each equipped with eight beds, provided care for non-COVID-19 patients admitted from the Emergency Department, operating rooms, or other hospital wards. At the time of the study, the nurse-to-patient ratio was 1:2. The length of stay (LOS) was, on average, 5 days, and the occupancy bed rate was 80%. Before the COVID-19 pandemic, these ICUs adopted a flexible visiting policy, with an open family presence at the bedside, while during the first wave [14] visitors have been prohibited from entering facilities. From the end of the first wave of the pandemic (June 2020) to October 21st, 2020, one relative/at a time was allowed to visit their loved ones one hour a day. During the second and third wave, from October 2020 to June 2021, a ‘no visitor’ policy has been applied.

A purposive sampling [23] was used to include one relative of each adult patient (≥ 18 years) with a LOS ≥ three days cared for in ICU from August 15th to October 20th, 2020. Inclusion criteria were (a) being a wife/husband, or being a blood relation of the patient, or being the identified next of kin; (b) being of the age of 18 or above; and (c) being willing to visit the patient daily. Due to the range of relationships possible in the above-mentioned criteria, all those who agreed to participate have been referred to as “relatives”. There were excluded (a) relatives of patients diagnosed as terminally ill, and (b) those with an unexpected suspension of visiting their loved ones in ICU daily. When the ICU family visits have been suspended, the recruitment was ended at this point according to the pragmatic impossibility to involve further relatives; however, the data saturation [24] was reached as judged independently by two researchers (MD, ST). According to the data saturation ascertained, no further participant involvement was provided as also suggested by the evidence available [24].

### Data collection

Relatives meeting inclusion criteria were approached for the study by a member of the research team within the first 24 h of the ICU admission. The informed consent form was written in the Italian language, using a vocabulary easily understandable by all potential participants; moreover, given the stressful nature of the experience lived by eligible relatives, the information regarding the study aims and procedures was given by an expert ICU nurse, in a calm environment, when it was appropriate. All relatives were given 24 h to consider their participation in the study.

After three months following ICU discharge, relatives’ experience was collected through telephone interviews, as participants were not authorised to entry in the hospital, or researchers were limited in conducting face-to-face interviews due to the pandemic restrictions. The interviews occurred between November 2020 and January 2021, with the last achieved on January 30th. One researcher (MD, Ph.D. student, Nurse Educator expert in ICU care, and not involved in the care of patients at the time of the study) performed all calls and conducted the audio-recorded interviews. The overall mean interview length was 28 min and ranged from 10 to 40 min. The interviews were conducted using an interview guide including closed and open-ended questions (see Supplementary Table 2). Each interview started with an open-ended question to introduce the discussion: “*Please, tell me about your experience just after the ICU discharge up to now*”. Then, the following main question was introduced: ‘*Can you please share your experience about caring for your beloved and the challenges you have encountered in daily life since the ICU discharge?*’. Moreover, probing questions were asked during the call (e.g., ‘*What do you mean?*’ and ‘*Can you explain this concept a little further?*’) to clarify the experiences or turn the attention back to the main topic. Participants were encouraged to share as much of their experience as possible. The questions were not provided in advance to the relatives.

### Data analysis

Quantitative data were entered into a Microsoft Excel® worksheet. While continuous variables (e.g., age, LOS) were displayed as mean, standard deviations (SD) and median, nominal variables (e.g., gender, the reason for admission) were shown as absolute frequencies and percentages.

All interviews were audio-recorded and transcribed verbatim. Then, the data were thematically analysed in the following inductive steps [25]. The first step was *achieving familiarity with the data* through open‐minded reading. Three researchers (MD, AP, AD) conducted a careful and thorough reading to obtain a comprehensive view of the experiences lived by participants paying attention to the words used by each relative. Researchers also identified and labelled representative quotations. Thereafter, the *search for meanings* was deepened. A preliminary data coding was performed by the same three researchers independently by generating a total of 18 initial codes whereby each quotation extracted was categorised. Lastly, the researchers *reached a meaningful wholeness*. Thus, codes with similar meanings and concepts were grouped into eight categories. Researchers discussed the findings that emerged and labelled three themes through a constructive dialogue. A few disagreements emerged among researchers, and all regarded unclear definitions given due to the different interpretations performed (e.g., ‘searching for support in providing care’ instead of ‘being supported in providing care’). These disagreements were solved by consulting a fourth researcher (ST).

### Validity and rigor

Credibility, dependability, confirmability and transferability [26] were ensured. First, credibility in the data and the findings was ensured by waiting for three months from the ICU discharge before contacting relatives. This was essential to reach an in-depth understanding of the care experience [4, 5, 8]. Second, dependability was guaranteed with a rich description of the study method, which allows the qualitative inquiry to be repeatable. Third, confirmability was reached by ensuring agreement among researchers, who independently analysed the data and agreed upon the findings. Transferability has been established by providing patient and relative profiles, thus allowing the evaluation of the findings in their validity in other contexts, situations, times and populations.

### Ethics

The study protocol was approved by the Regional Ethics Committee of Friuli Venezia Giulia, Italy (CEUR-2020-Sper-012) and conducted according to the criteria set by the Declaration of Helsinki; moreover, each relative provided written informed consent after having received appropriate information regarding the research aims and procedures. Since critically ill patients were under life-threatening conditions, the ICU team was involved aiming at ensuring that the informed consent was collected according to the situation and when appropriate in the patient and relative best interest. Confidentiality was ensured by the researchers during each data handling process. In reporting findings, relatives’ identity was protected: specifically, quotations were indexed as being from a family member interviewed, numbered consecutively (e.g., R1, Relative number 1). In addition, relatives were free to withdraw from the study at any time without the need to provide reasons.

## Results

### Participants

**Fig 1.**
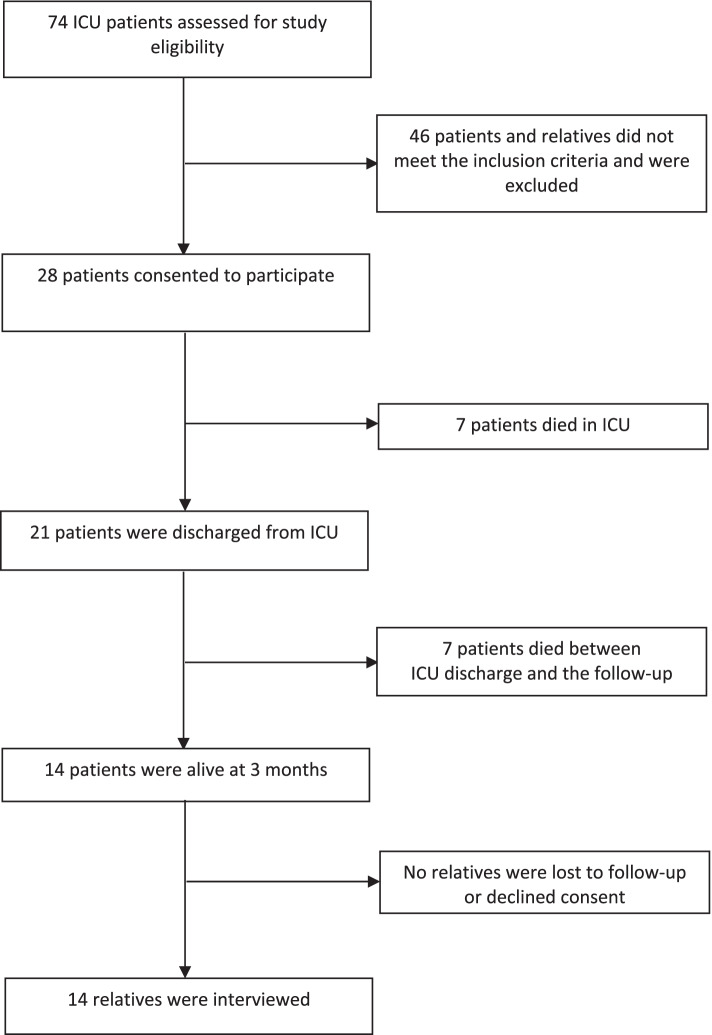
Flowchart of patient recruitment and follow-up

A total of 28 patient–family member pairs were enrolled (Fig. 1), from whom baseline data were collected; 21 (75.0%) patients survived and were discharged from the ICU and 14 (50.0%) were alive at the three-month follow-up.

Survivors (Table 1) were predominantly males (*n* = 11; 78.6%), with an average age of 59.4 years (SD 16.0, median 70). They had been admitted in ICU following organ failure, trauma, or cerebrovascular disease (*n* = 6; 42.8%, *n* = 6; 42.8%, and *n* = 2; 14.4%, respectively). The average LOS was 18 days (SD 9.0, median 15). As reported in Table 2, relatives were mainly the partner (e.g., spouses) (*n* = 7; 50.0%), females (*n* = 11; 78.6%), with an average age of 53.9 years (SD 9.5, median 51), and with the majority (*n* = 9, 64.3%) being secondary school graduates. Most of the participants (*n* = 11, 78.5%) were employed either in the public or in the private sector, with 21.5% (*n* = 3) not in workforce (e.g., unemployed, retired). The majority of the participants reported living with the patient (*n *= 8, 57.1%).

**Table 1:** Baseline and three-month follow-up socio-demographic characteristics of included patients

	**Baseline** ***n* = 28**	**Three-month follow-up** ***n* = 14**
Age (years), mean (SD; median)	67.3 (15.0; 72)	59.4 (16.0; 70)
Gender, n (%)		
Female	7 (25.0)	3 (21.4)
Male	21 (75.0)	11 (78.6)
Reason for ICU admission, n (%)		
Organ failure	17 (60.7)	6 (42.8)
Trauma	7 (25.0)	6 (42.8)
Cerebrovascular disease	3 (10.7)	2 (14.4)
Post-operative	1 (3.6)	-
At least one comorbidity, n (%)	17 (60.7)	8 (57.1)
Length of stay in ICU (days), mean (SD; median)	15.0 (8.0; 15)	18.0 (9.0; 15)

*SD S*tandard deviation, *ICU I*ntensive care unit

**Table 2:** Baseline and three-month follow-up socio-demographic characteristics of relatives

	**Baseline** ***n* = 28**	**Three-month follow-up** ***n* = 14**
Age (years), mean (SD; median)	55.4 (10.9; 50)	53.9 (9.5; 51)
Gender, n (%)		
Female	23 (82.1)	11 (78.6)
Male	5 (17.9)	3 (21.4)
Relationship to patient, n (%)		
Spouse/husband or significant partner	13 (46.5)	7 (50.0)
Daughter/son	10 (35.7)	3 (21.4)
Mother/father	2 (7.1)	2 (14.4)
Sister/brother	1 (3.6)	1 (7.1)
Other degree of relatedness	2 (7.1)	1 (7.1)
Education, n (%)		
Primary school	7 (25.0)	4 (28.6)
Secondary school	15 (53.6)	9 (64.3)
Degree or above	6 (21.4)	1 (7.1)
Employment, n (%)		
None	4 (14.3)	2 (14.4)
Public employee	8 (28.6)	5 (35.7)
Private employee	10 (35.7)	6 (42.8)
Retired	6 (21.4)	1 (7.1)
Prior experience with ICU, n (%)	8 (28.6)	4 (28.6)
Cohabitation with the patient, n (%)	13 (46.4)	8 (57.1)
How often the relative was seeing the patient, n (%)		
More than weekly	20 (71.4)	12 (85.6)
Weekly	7 (25.0)	2 (14.4)
Monthly	1 (3.6)	-

*SD S*tandard deviation, *ICU I*ntensive care unit

### The lived experience

Three main themes emerged, consisting of eight categories (Table 3), namely “Being shaken following the ICU discharge”, “Returning to our life that is no longer the same”, and “Feeling powerless due to the COVID-19 pandemic”.

**Table 3:** Data synthesis by extracting and abstracting findings in common categories and themes

**Abstraction: Themes**	**Abstraction: Categories**	**Codes as defined by researchers**	**Example of quotations extracted from interviews**
Being shaken following the ICU discharge	Experiencing negative feelings	Astonishment at unexpected news	‘*Bad, really bad, no life expectancy... I remember these words, as if they were imprinted with fire’* [R1]
		Despair for what has happened	‘*The situation is challenging. As relatives, we all suffer with him, every day*’ [R1]
		Anger regarding conflicting information	‘*In ICU I was told that the tracheostomy was going to be removed. In the ward, however, I was told that she would never swallow or eat again’* [R14]
		Fear of complications arising	‘*I just hope the lung infection doesn’t come back again…he wouldn’t survive another illness’* [R9]
	Experiencing positive feelings	Trust in healthcare professionals	‘*I have to fully rely on doctors and nurses, also because I’m not an expert and so I have to trust them’* [R1]
		Gratitude for the care provided	‘*In my opinion he has always been well looked after. I have nothing to say about the care they gave him, I’m just grateful’* [R2]
Returning to our life that is no longer the same	Realising that nothing can be as before	Awareness of the limitations	“*I haven't seen him for two months; I can only bring him his clothes once a week and leave them outside the ward*” [R9] “*He doesn't walk, he doesn't move by himself, he can't even sign. We're doing the paperwork for the disability pension*” [R2] “*For her to eat, everything has to be blended now, there is little to do, that's it*” [R14] “*An example: she is no longer able to cook pasta*” [R4]
		Rearrange the home environment	“*I got a stationary bike to allow him to do some exercise. He cannot still climb the stairs*” [R9]
	Searching for support in providing care	Benefit from community-based services	“*At home, we are followed by home care, a nurse visits us. If there are any critical issues, we ask her*” [R13]
		Enlist privately hired professionals	“*As to rehabilitation, I opted for a private service. I called a physiotherapist who is now helping us*” [R13] “*We’ve arranged a physiotherapist and a caregiver*” [R3] “*A private nurse comes twice a day*” [R13]
	Changing my life	Change my routine	“*At first I ate what she ate, so as not to give her cravings*” [R14] ‘*When we got home, I used to sleep in the living room so as not to stay too close and bring bacteria or viruses near her’* [R8]
		Balance caring with other activities	‘*I had to take time off work to assist her’* [R10] ‘*I also have to get someone to do grocery shopping so that I don’t leave her alone’* [R11]
Feeling powerless due to the COVID-19 pandemic	Altering the clinical pathway	Early discharge from rehabilitation structure	‘*It was a hard blow for her when she was discharged from the rehabilitation hospital – she needed one more month’* [R3]
		Access denied to long-term care	‘*She is still hospitalized due to COVID-19, she is not accepted in any other facility. I applied but we are still on hold’* [R4] ‘*The rehabilitation facility has fewer beds now ... neurorehabilitation, which she would need, is now occupied by COVID-19 patients’* [R7]
		Regular check-ups cancelled	‘*The first visit with the doctor was put off due to COVID-19. In January, the neurological check was cancelled as well’*” [R10]
	Restricting family and friends visiting	Patients’ loneliness	‘*If she had any friends, someone who could stimulate her…’* [R7] ‘*Some colleagues and friends of hers came to the house, but only to the door, no further than that’* [R8]
		Missed carer training programme	‘*We relatives should have had some training, but it was limited’* [R13]
	Thinking about the future	Make important choices	‘*Where to place him is going to be a problem because he used to live on the second floor ... a more equipped facility is needed ... I think I will opt for a nursing home’* [R2] ‘*I thought that a facility was going to be the right option for her ... she would be looked after by people who are not just company but competent’* [R4]

*R1 *Relative n.1, *ICU *Intensive Care Unit, *COVID-19 *coronavirus disease 2019.

### Theme 1: Being shaken following the ICU discharge

Relatives reported having felt a sense of astonishment in listening to the unexpected news at the ICU discharge: ‘*Bad, really bad, no life expectancy…I remember these words as if they were imprinted with fire’* [R1]. This emotion triggered a widespread sense of despair for what was happening: ‘*The situation is challenging. As relatives, we all suffer with him, every day’* [R1]. Negative feelings such as anger towards healthcare professionals were also reported when conflicting information was given from one department to another: ‘*In ICU I was told that the tracheostomy was going to be removed. In the ward, however, I was told that she would never swallow or eat agai*n’ [R14]”. Relatives also experienced fear of possible complications: ‘*I just hope the lung infection doesn’t come back again…he wouldn’t survive another illness’* [R9].

By contrast, relatives reported their positive experiences as trusting in healthcare professionals who they saw as experts and competent in managing the conditions and in giving information regarding the future: ‘*I have to fully rely on doctors and nurses, also because I’m not an expert and so I have to trust them’* [R1]; moreover, they also reported the gratitude for the care provided to their loved one: ‘*In my opinion, he has always been well looked after. I have nothing to say about the care they gave him, I’m just grateful’* [R2].

### Theme 2: Returning to our life that is no longer the same

Participants become aware of the limitations in the activities of the daily life of their loved one: ‘*He does not walk, he does not move by himself, he can't even sign. We’re doing the paperwork for the disability pension’* [R2]. As a consequence, relatives rapidly changed the physical environment of their houses, according to the specific limitations and also in the attempt to maximise the patient’s functional independence: ‘*I got a stationary bike to allow him to do some exercise. He cannot climb the stairs anymore’* [R9].

Participants felt supported, having benefited from community-based services: ‘*At home, we are followed by home care, a nurse visits us. If there are any critical issues, we ask her’* [R13]. However, they focused their priorities on hiring private professionals, as in the case of physiotherapists: ‘*As to rehabilitation, I opted for a private service. I called a physiotherapist who is now helping us’* [R13] to accelerate the healing or the rehabilitation process, but also to compensate for the lack of care offered by the community health services available: “*We’ve arranged a physiotherapist and a caregiver*” [R3].

The complexity of the experience also affected the life of the relatives. They reported changing their routines, as in the case of sleeping habits: ‘*When we got home, I used to sleep in the living room so as not to stay too close and bring bacteria or viruses near her’* [R8]. To protect themselves, relatives reported the need to balance the caring role with the multiple roles lived before: ‘*I also have to get someone to do grocery shopping so that I don’t leave her alone’* [R11].

### Theme 3: Feeling powerless due to the COVID-19 pandemic

Participants felt they lacked the power to ensure the full professional care required on the one hand, and also the required emotional support offered by informal carers, such as friends, on the other. In addition, they also perceived themselves as being unprepared to play the complex carer role required.

Some relatives felt that patients have been discharged from the rehabilitation units too early: ‘*It was a hard blow for her when she was discharged from the rehabilitation hospital – she needed one more month’* [R3]. In contrast, others were unable to receive care in long-term care facilities, which have been converted into COVID-19 units: ‘*The rehabilitation facility has fewer beds now…neurorehabilitation, which she would need, is now occupied by COVID-19 patients’* [R7]. Moreover, the COVID-19 pandemic has also prevented the regular check-ups, which are often cancelled or delayed: ‘*The first visit with the doctor was put off due to COVID-19. In January, the neurological check was cancelled as well’* [R10].

The isolating environment imposed by the COVID-19 pandemic, restricting family and friends’ visits, led to patients’ loneliness, as reported by relatives: ‘*If she had many friends, someone who could stimulate her*…’ [R7]. In addition, carers’ training programs were often missed or reduced in duration: ‘*We relatives should have had some training, but it was limited’* [R13]. The sense of powerlessness and the lack of available support mean that relatives have to start thinking about the future and the important choices required: ‘*I thought that a facility was going to be the right option for her … she would be looked after by people who are not just company but competent*’ [R4].

## Discussion

To the best of our knowledge, this is the first study exploring the experiences of relatives of critically ill patients discharged from non-COVID ICUs during the COVID-19 pandemic. Three main themes emerged: “Being shaken following the ICU discharge”; “Returning to our life that is no longer the same” and “Feeling powerless due to the COVID-19 pandemic”. While the first two themes are substantially in line with previous evidence [4–6], the third theme highlights important findings and discussion points, suggesting the need to focus on family caregivers and how to include them in pandemic and post-pandemic clinical decision-making [27]. In addition, as patients’ quality of life deterioration may have a serious impact also on relatives, post-ICU discharge implications on the care needs of the dyad (patient + relative) should be investigated further [28]. Restrictive policies did not allow relatives to be engaged in the care of their loved ones during the in-hospital stay; they have also experienced difficulties at home in receiving visits and support, due to restrictions imposed by the COVID-19 pandemic responses at the community level.

### The lived experience of relatives

In the first stage of the experience, when discharged from the ICU, relatives lived a twofold emotional condition. They felt despair and concerns regarding their beloved, findings consistent with those that emerging from a two-month follow-up study involving 115 US carers where the psychological burden (e.g. sadness, distress, anxiety) has been reported [29]. Negative feelings were also aroused when relatives received conflicting information. As previously documented, the negative psychological impact on relatives is exacerbated when they do not receive sufficient support and consistent information from healthcare professionals [30]. However, while some of these emotions are unavoidable, such as being surprised and conflicted by what has happened, others (e.g., anger, fear) might be prevented by appropriate support.

On the other hand, relatives reported trusting the ICU staff and expressed gratitude for their work, suggesting that also in hard working conditions and barriers to effective communication that have been limited to video calls, healthcare professionals have been perceived as compassionate and effective in the care delivered. Conversely, no concerns for the relatives’ physical health emerged at this stage, according to the findings.

The return to the daily routine implied a sort of invention of a new life: relatives immediately acquired awareness of the limitations of their beloved in the daily activities and the need to rearrange the home environment [5], to adapt the traditional house to the new needs, a process that requires additional expenses. Relatives seem to realise these limitations while at home and to find solutions in the lived moment, without being prepared: this unpreparedness might be a consequence of the absence of engagement during the in-hospital length of stay.

Searching for and obtaining support from the community services has also been reported by relatives. As described in a cross-sectional study on 157 family carers in South Korea, home care services, such as home-visit nursing or bathing, have been documented as positively impacting healthy family functioning [31]. However, as not all these services are available, family carers reported having paid out of their pocket to obtain such services. Additionally, the care daily required to impose a change in the relative’s life and the need to accommodate the different roles and responsibilities played in their life. A systematic review on informal carers of ICU survivors involved in their care reported that up to 50% of them reduced their work hours, quit their job or had been fired [8]. These effects on jobs should be read in light of the economic crisis triggered by the COVID-19 pandemic and of the additional costs required by the care at home, suggesting that relatives of ICU-discharged patients are at risk of acquired poverty that might affect their lives and also that of their loved one.

The COVID-19 pandemic has affected ICU accessibility and relatives’ engagement, with negative influences also at the time of discharge, making them unable to manage the complex conditions. On the other hand, the priority given to COVID-19 patients and units has affected the availability of healthcare opportunities (e.g. home health assistance, follow-up visits) [27]. In addition, the pandemic has dramatically altered nearly every aspect of their life, including the most basic social interactions, forcing the person to be confined at home [32]. Therefore, relatives seem to have experienced a bilateral restriction of opportunities, both at the hospital and at the home level, regarding both formal and informal care. Additionally, the minimisation of contact with healthcare professionals at the hospital and the community level seems to have impaired the quality of care, suggesting that a strong investment in non-COVID-19 patients discharged from the hospital should be considered a priority. The risk of having avoidable admissions in nursing homes is high, as reported by relatives as a consequence of the care burden, which is also due to the poor support received, as documented before the COVID-19 pandemic [31].

The lived experiences of relatives that have emerged, suggest nurses design and implement strategies (a) ensuring relatives’ in-hospital engagement and training programmes to overcome their unpreparedness while at home, by observing all COVID-19 preventive measures; (b) providing ICU follow-up also for relatives, to early detect specific needs; and (c) assessing the relatives mental and physical wellbeing, given that their health and quality of life are essential also for the clinical outcomes of their beloved. The consequences of the COVID-19 pandemic are international as lockdowns have increased anxiety and have reduced resilience among relatives, also due to the limitations imposed by the restrictions on hospital visiting. Continuing to investigate their experience in short- and long- terms also in other countries, might increase the evidence available and inform policies tailored for non-COVID-19 ICUs that have been exposed to the same restrictions as the COVID-19 ICUs.

### Limitations

The study has several limitations. First, participants’ recruitment was influenced by the changes in the health care services that occurred in the second wave of the COVID-19 pandemic that began in Autumn 2020, since a ‘no visitor’ policy has been applied in ICU. Second, the three-month follow-up should be considered a short-term to investigate the complexity of the experience lived by ICU survivors’ family members: therefore, there is a need to further investigate the long-term experiences of relatives. Third, the data collection has been performed via telephone, and this might have prevented in-depth sharing of the experiences as lived by relatives, in addition to the absence of visual cues, contextual and nonverbal data that might have compromised an effective data collection.

### Implications of clinical practice

During the ongoing COVID-19 pandemic, healthcare services should give priority to discharged patients in an attempt to compensate for the missed care. When possible, relatives need to be provided with appropriate hands-on training or education before discharge. Up to the ICUs reopening to external visits, relatives should be supported with alternative solutions, such as telephone consultations with nurses and physicians, professional online support and follow-up. The individual experiences trigger urgent public health considerations to prevent patient and relative’s mental and physical health issues.

## Conclusion

Relatives of critically ill patients discharged from a non-COVID-19 ICU experienced a mix of negative and positive feelings in the early stages. Once at home, the limitations of the community services available have triggered the search for additional support by private healthcare providers. Moreover, some issues of care have been exacerbated by altering the clinical pathway of the patient and forcing the dyad – the patient and his/her relative, toward loneliness. Relatives seem to have experienced a bilateral restriction of opportunities, both at the hospital, without any engagement and with limited possibility to access the ICU, and at the home level in terms of formal and informal care.

## Supplementary Information

**Additional file 1: Supplementary Table 1.** COnsolidated criteria forREporting Qualitative research checklist. **Supplementary Table 2**. Interview guide. 

## Data Availability

The full-text interviews collected during the study are not publicly available as not in English language (Italian) but are accessible from the corresponding author on reasonable request.
