# Supplementary material for: Experience of relatives in the first three months after a non-COVID-19 Intensive Care Unit discharge: a qualitative study
Source: BMC Prim Care. 2022 May 5;23:105. doi: 10.1186/s12875-022-01720-z (PMC9071510; doi:10.1186/s12875-022-01720-z)
Supplement: Supplementary file 1 — Additional file 1: Supplementary Table 1. COnsolidated criteria forREporting Qualitative research checklist. Supplementary Table 2. Interview guide. [file 12875_2022_1720_MOESM1_ESM.docx]

**SUPPLEMENTARY TABLE 1** COnsolidated criteria for REporting Qualitative research checklist

| **Topic** | **Item No.** | **Guide Questions/Description** | **Reported on** |
| --- | --- | --- | --- |
| **Domain 1: Research Team and Reflexivity** | | | |
| *Personal Characteristics* | | | |
| Interviewer/facilitator | 1 | Which author/s conducted the interview or focus group? | *Data collection* |
| Credentials | 2 | What were the researcher’s credentials? E.g. PhD, MD | *Data collection* |
| Occupation | 3 | What was their occupation at the time of the study? | *Data collection* |
| Gender | 4 | Was the researcher male or female? | *Data collection* |
| Experience and training | 5 | What experience or training did the researcher have? | *Data collection* |
| *Relationship with Participants* | | | |
| Relationship established | 6 | Was a relationship established prior to study commencement? | *Data collection* |
| Participant knowledge of the interviewer | 7 | What did the participants know about the researcher? e.g. personal  goals, reasons for doing the research | *Data collection* |
| Interviewer characteristics | 8 | What characteristics were reported about the inter viewer/facilitator?  e.g. Bias, assumptions, reasons and interests in the research topic | *Validity and rigor* |
| **Domain 2: Study Design** | | | |
| *Theoretical Framework* | | | |
| Methodological orientation and Theory | 9 | What methodological orientation was stated to underpin the study?  e.g. grounded theory, discourse analysis, ethnography, phenomenology, content analysis | *Background and design* |
| *Participant Characteristics* | | | |
| Sampling | 10 | How were participants selected? e.g. purposive, convenience,  consecutive, snowball | *Setting and participants* |
| Method of approach | 11 | How were participants approached? e.g. face-to-face, telephone, email | *Setting and participants* |
| Sample size | 12 | How many participants were in the study? | *Setting and participants* |
| Non-participation | 13 | How many people refused to participate or dropped out? Reasons? | N/A |
| *Setting* | | | |
| Setting of data collection | 14 | Where was the data collected? e.g. home, clinic, workplace | *Data collection* |
| Presence of non-  participants | 15 | Was anyone else present besides the participants and researchers? | *Data collection* |
| Description of sample | 16 | What are the important characteristics of the sample? e.g. demographic data | *Table 1-2* |
| *Data Collection* | | | |
| Interview guide | 17 | Were questions, prompts, guides provided by the authors? Was it pilot tested? | *Data collection* |
| Repeat interviews | 18 | Were repeat inter views carried out? If yes, how many? | N/A |
| Audio/visual recording | 19 | Did the research use audio or visual recording to collect the data? | *Data collection* |
| Field notes | 20 | Were field notes made during and/or after the interview or focus group? | *Data collection* |
| Duration | 21 | What was the duration of the inter views or focus group? | *Data collection* |
| Data saturation | 22 | Was data saturation discussed? | *Limitations* |
| Transcripts returned | 23 | Were transcripts returned to participants for comment and/or correction? | N/A |

| **Topic** | **Item No.** | **Guide Questions/Description** | **Reported on** |
| --- | --- | --- | --- |
| **Domain 3: analysis and findings** | | | |
| *Data analysis* | | | |
| Number of data coders | 24 | How many data coders coded the data? | *Data analysis* |
| Description of the coding tree | 25 | Did authors provide a description of the coding tree? | *Data analysis and*  *Table 3* |
| Derivation of themes | 26 | Were themes identified in advance or derived from the data? | *Data analysis and Table 3* |
| Software | 27 | What software, if applicable, was used to manage the data? | N/A |
| Participant checking | 28 | Did participants provide feedback on the findings? | N/A |
| *Reporting* | | | |
| Quotations presented | 29 | Were participant quotations presented to illustrate the themes/findings?  Was each quotation identified? e.g. participant number | *Results and Table 3* |
| Data and findings consistent | 30 | Was there consistency between the data presented and the findings? | *Results, Table 3, Discussion* |
| Clarity of major themes | 31 | Were major themes clearly presented in the findings? | *Results and Table 3* |
| Clarity of minor themes | 32 | Is there a description of diverse cases or discussion of minor themes? | *Results and Table 3* |

N/A = not applicable.

**SUPPLEMENTARY TABLE 2** Interview guide

| **Close questions**  (1) Age, (2) gender, (3) relationship to patient (e.g., spouse/husband), (4) education (e.g., primary/secondary school, degree level), (5) work status (retired, employed); (6) prior experience with ICU (yes/no); (7) living together with the patient (yes/no), (8) how often the relative visited the patient (e.g., weekly).  **Open-ended questions**  9) Please, tell me about your experience just after the ICU discharge up to now.  10) Can you please share your experience about caring for your beloved and challenges you have encountered in daily life since the ICU discharge?  11) Which emotions did you live at the point of ICU discharge up to now?  12) What did the ICU discharge mean to you, as family member?  13) How would you describe the ideal care/support at this stage of everyday care?  14) Do you want to add something?  **Interview conclusion**  At the end of the interview: the interviewer reformulated the main contents/emotions shared by the family member. |
| --- |

ICU = intensive care unit.
